# Borderline Personality and the Detection of Angry Faces

**DOI:** 10.1371/journal.pone.0152947

**Published:** 2016-03-31

**Authors:** Johanna Hepp, Benjamin E. Hilbig, Pascal J. Kieslich, Julia Herzog, Stefanie Lis, Christian Schmahl, Inga Niedtfeld

**Affiliations:** 1 Department of Psychosomatic Medicine, Central Institute of Mental Health, Medical Faculty Mannheim/ Heidelberg University, Mannheim, Germany; 2 Department of Psychology, University of Koblenz-Landau, Landau, Germany; 3 Department of Psychology, University of Mannheim, Mannheim, Germany; University of Tasmania, AUSTRALIA

## Abstract

**Background:**

Many studies have assessed emotion recognition in patients with Borderline Personality Disorder and considerable evidence has been accumulated on patients’ ability to categorize emotions. In contrast, their ability to detect emotions has been investigated sparsely. The only two studies that assessed emotion detection abilities found contradictory evidence on patients’ ability to detect angry faces.

**Methods:**

To clarify whether patients with Borderline Personality Disorder show enhanced detection of angry faces, we conducted three experiments: a laboratory study (n = 53) with a clinical sample and two highly powered web studies that measured Borderline features (n_1_ = 342, n_2_ = 220). Participants in all studies completed a visual search paradigm, and the reaction times for the detection of angry vs. happy faces were measured.

**Results:**

Consistently, data spoke against enhanced detection of angry faces in the Borderline groups, indicated by non-significant group (Borderline vs. healthy control) × target (angry vs. happy) interactions, despite highly satisfactory statistical power to detect even small effects.

**Conclusions:**

In contrast to emotion categorization, emotion detection appears to be intact in patients with Borderline Personality Disorder and individuals high in Borderline features. The importance of distinguishing between these two processes in future studies is discussed.

## Introduction

Borderline Personality disorder (BPD) is a severe psychiatric disorder that affects around 1 to 3% of the adult population and is characterized by marked affective instability, impulsivity, and interpersonal problems [1, 2]. Dysfunctional emotion processing is considered one of the central factors that contribute to the phenomenology of this disorder. As a first step in the emotion processing continuum, the question of how well BPD patients can recognize emotions in others has gained particular interest in the past [3]. Numerous studies on emotion recognition in BPD have been conducted and finally summarized in two comprehensive literature reviews [4, 5] as well as two recent meta-analyses [6, 7]. We argue that the studies summarized therein actually capture two distinct aspects of emotion recognition, namely emotion categorization and emotion detection, and present new evidence on the relatively under-researched aspect of emotion detection.

According to a recent series of experiments, emotion categorization and detection are distinct processes that require distinct abilities and should thus be discriminated from each other [8]. Emotion categorization requires the ability to correctly identify and verbally label a presented emotion. In previous studies on BPD samples, this ability was measured by asking participants to indicate which emotion they saw in a face by choosing the correct term from a set of pre-defined answering options. A meta-analysis on these studies revealed an emotion categorization deficit across all emotions and selective deficits for differentiating between disgust and anger [5].

In contrast, emotion detection does not require specific emotions to be named and distinguished from one another; it simply comprises the ability to accurately perceive whether *any* emotional content is present in a face or not. Emotion detection has previously been captured using morphing paradigms in which an initially neutral face gradually changes into an emotional one. The earliest stage in the morphing process at which participants perceive some degree of emotion in the face (and therefore stopped the morphing process) thus indicates their emotion detection abilities. Evidence on BPD patients’ performance in this task was mixed. Two studies found no difference regarding emotion detection between healthy controls (HC) and BPD patients [9] or youth with BPD symptoms [10]. Others found superior [11] and still others found inferior [12] emotion detection performance in the included BPD groups. Finally, high BPD symptom counts predicted a lower detection threshold for angry male faces in a non-patient sample [13]. Evidently, clear conclusions regarding BPD patients’ emotion detection abilities are not possible based on this body of work. One potential problem with morphing studies is that they assess not only emotion detection but also an additional element of emotion categorization, because, once the morphing process is stopped, participants still have to label the displayed emotion. To gain a better understanding of BPD patients’ emotion detection abilities it thus seems necessary to assess detection in paradigms that do not also include categorization.

So far, only two studies have addressed emotion detection outside of morphing studies. The first study by Schulze and colleagues used a rapid, continuous stream of stimuli consisting of facial photographs that were cropped into a standard oval shape [14]. Several trials included a happy or angry face, whereas others included only neutral looking faces. After each trial, participants indicated whether an emotional face was present or not. No main effect of group was found, but BPD patients were selectively more sensitive towards angry stimuli, producing greater hitrates for angry faces than the HC group. A second study by Hagenhoff and colleagues [15] assessed emotion detection abilities for happy and angry faces using the face-in-the-crowd-paradigm [16]. In this paradigm, emotional faces have to be detected in “crowds” (i.e. matrices) of neutral faces. In the past, the face-in-the-crowd paradigm has repeatedly revealed an anger superiority effect (ASE): angry faces in neutral crowds were detected faster and with fewer errors than happy faces in neutral crowds [17]. Hagenhoff and colleagues employed line drawings of round faces (“smiley faces”) and analyzed the reaction times with which participants were able to detect angry faces. They found no overall group difference but also no detection advantage for angry faces in the BPD group, which stands in contrast to the previous findings by Schulze and colleagues.

In sum, while there is considerable previous evidence on emotion categorization in BPD [6, 7], evidence on emotion detection is sparse. Moreover, the only two existing studies that have used paradigms that assess emotion detection directly found contradicting evidence for the angry condition [14, 15]. Schulze and colleagues found an increased sensitivity for angry faces while Hagenhoff and colleagues found no specific advantage for angry faces. As a result of this, it remains entirely unclear whether patients’ detection abilities are in fact altered, particularly for angry faces, and further studies are clearly needed to resolve this.

In an effort to shed further light on this topic we conducted a detection-only study. In line with the procedure of the two previous detection studies, we focused on happy and angry stimuli. Specifically, we chose to use a face-in-the-crowd-paradigm similar to the one Hagenhoff and colleagues used. We preferred this paradigm to the one employed by Schulze and colleagues because it is a more immediate measure of emotion detection: in the face-in-the-crowd paradigm participants respond immediately to a detected face, whereas in the other paradigm participants indicate whether they have detected an emotional face with delay, at the end of a picture sequence. For the face-in-the-crowd paradigm, we intended to improve upon previous material by creating a new set of geometrically controlled facial stimuli that would minimize confounding visual effects: when happy stimuli are drawn such that eyebrows and mouth are parallel to the facial surround, this can cause greater sensitivity for the angry faces simply because these are more salient [18]. In line with previous studies using the face-in-the-crowd paradigm, we expected that (1) BPD and HC participants would all show an ASE regarding reaction times, thus detecting angry faces faster than happy faces. Additionally we assessed whether using geometrically controlled stimuli (2) BPD patients would show an anger detection advantage in the form of a stronger ASE in the face-in-the-crowd paradigm, mirroring the one previously found by Schulze and colleagues.

## Methods Study 1

### Participants

Twenty-nine female BPD patients and 28 female HCs were recruited between May 2011 and December 2011. Patients were recruited through a patient database at the Central Institute of Mental Health, Mannheim, Germany, and healthy control participants were recruited through advertisements in local newspapers After applying a criterion for a minimal usable trial number of 90% per condition, twenty-seven participants remained in the BPD group and 26 participants comprised the control group. BPD patients were diagnosed by an experienced clinician using the German version of the structured clinical interview for the DSM-IV [19] and the International Personality Disorder Examination [20]. General exclusion criteria were comorbid diagnoses of schizophrenia, bipolar disorder, developmental disorder, or substance dependency. The majority of patients were unmedicated (61.5%); several took antidepressants (19.2%), neuroleptics (7.7%), or a combination of both (11.5%). Patients had on average 1.9 comorbid disorders (SD = 1.5), which included mainly anxiety disorders (n = 19), mood disorders (n = 12), eating disorders (n = 4), other personality disorders (n = 3), and each one case of pain disorder, alcohol abuse, and trichotillomania. Patients mean score of 2.05 (SD = 0.84) on the Borderline-Symptom-List, indicated symptom severity similar to that observed in other patient samples [21]. Patients further had a mean score of 27.48 (SD = 10.14) on the Beck-Depression-Inventory [22], indicating clinically relevant depressive symptoms. Controls’ mean score of 0.37 on the Borderline-Symptom-List (SD = 0.41) and 5.27 (SD = 5.22) on the Beck-Depression-Inventory indicated the absence of clinically relevant BPD and depressive symptoms. Further, HC fulfilled no criteria for any present or past mental disorder. Patients (aged 18 to 42, M = 26.63, SD = 6.15) were matched to HC (aged 18 to 38, M = 25.19, SD = 5.43) for age and level of education.

### Material

**Fig 1:**
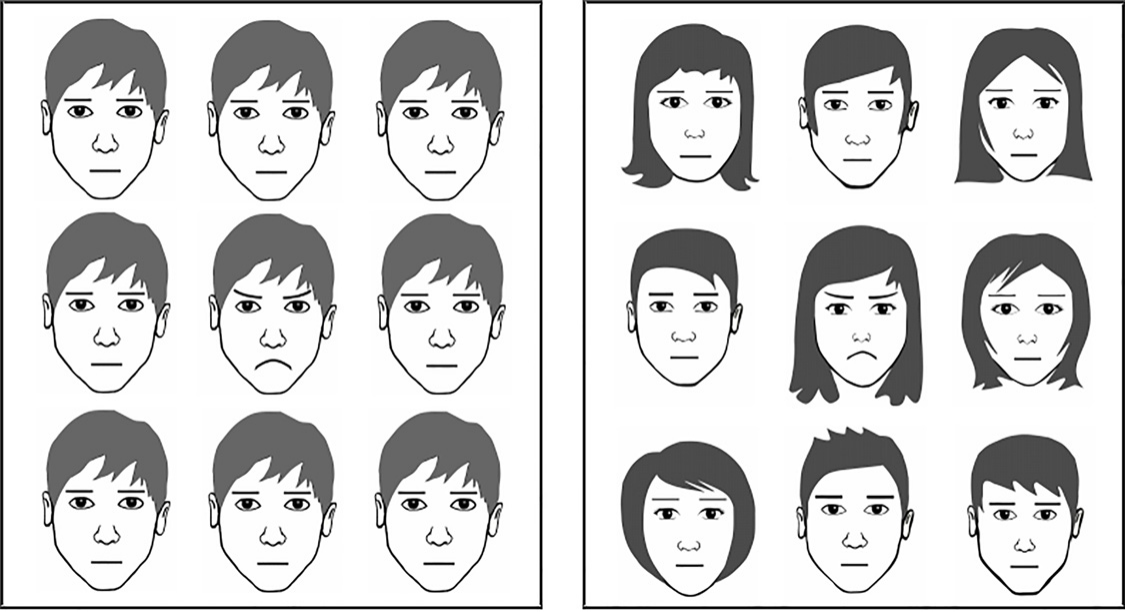
Exemplary face-in-the-crowd matrix with angry target for studies 1/2 (left) and study 3 (right).

In the face-in-the-crowd paradigm, each trail consisted of a 3×3 matrix of faces which followed a 1000ms inter-trail-interval. Participants were instructed to indicate as fast and accurately as possible whether a target face was present in the matrix (press A = “target present”, press L = “target absent”). Target faces included a happy, angry and a blue baseline stimulus. Stimuli were schematic faces designed using the vector drawing program Inkscape [23]. Importantly, angry and happy stimuli were geometrically controlled variants of the neutral face: Their eyebrows and mouth were exact mirror images of each other. Thus, in geometrical terms, they differed from the neutral face to exactly the same extent. Parallelism of eyebrows and facial surround was minimized by including hair. The blue stimulus, which served as a neutral baseline, was an exact copy of the neutral stimulus, but with blue eyebrows and mouth. An exemplary matrix with the stimuli included here is presented in Fig 1.

### Procedure

The study was reviewed and ethics approval was granted by the ethics committee of the University of Heidelberg before the study began (committee name: Medizinische Ethik-Kommission II der Medizinischen Fakultät Mannheim der Universität Heidelberg). At the beginning of the study all participants provided informed written consent after obtaining detailed information about the study procedure. The ethics committee approved of this procedure. Capacity to consent was determined by the patient’s diagnostician and was given for all participants. Next, participants provided demographical information and then filled in several questionnaires not pertinent to the current investigation. Next, participants completed four practice trials of the face-in-the-crowd paradigm, followed by a set of 216 experimental trials, consisting of 108 noise trials (no target) and 36 target trials for each type of target (angry/happy/blue).

## Results Study 1

The dataset for Study 1 can be accessed in S1 Dataset. Reaction times (in ms) were log-transformed due to non-normality. Extreme outliers with a reaction time more than 2.5 SD above the sample mean were excluded (1.2% of trials). All statistical analyses were conducted for correctly answered trials only (M = 93.6% of all trials, SD = 14.0%). All means, standard deviations and confidence intervals are presented separately for both groups in Table 1. Data was analyzed using a repeated measures ANOVA with reaction times as the dependent variable (see Table 2).

**Table 1:** Means (M), standard deviations (SD), and 95% confidence intervals (95%CI) of the mean (log-transformed) reaction times in all studies, presented for the different target types in the Borderline Personality Disorder group (BPD) and healthy control group (HC).

	HC		BPD	
target	M (SD)	95% CI	M (SD)	95% CI
**Study 1**				
angry	3.04 (0.09)	[3.03,3.06]	3.04 (0.07)	[3.03,3.06]
happy	3.10 (0.07)	[3.08,3.11]	3.10 (0.08)	[3.08,3.11]
blue	3.09 (0.08)	[3.07,3.10]	3.08 (0.07)	[3.06,3.10]
**Study 2**				
angry	3.01 (0.08)	[3.00,3.02]	3.04 (0.08)	[3.02,3.05]
happy	3.06 (0.09)	[3.05,3.07]	3.08 (0.08)	[3.07,3.09]
blue	3.05 (0.10)	[3.04,3.06]	3.09 (0.12)	[3.07,3.10]
**Study 3**				
angry	3.12 (0.08)	[3.11,3.13]	3.14 (0.08)	[3.13,3.15]
happy	3.15 (0.07)	[3.14,3.16]	3.18 (0.08)	[3.17,3.19]

**Table 2:** Test-statistics for the repeated measures ANOVA main effects of target type, group and their interaction for studies 1, 2, and 3.

Study	F	df	p	effect size f [90% CI]
**Main effect of target (within)**				
Study 1	33.45	(1.74, 88.52)	< .001	0.29 [0.21,0.36]
Study 2	92.94	(1.36, 461.65)	< .001	0.22 [0.18,0.25]
Study 3	124.72	(1, 218)	< .001	0.23 [0.19,0.27]
**Main effect of group (between)**				
Study 1	0.03	(1, 51)	.86	0.02 [0.00,0.19]
Study 2	8.70	(1, 340)	.003	0.15 [0.06,0.23]
Study 3	6.35	(1, 218)	.012	0.16 [0.05,0.27]
**Interaction effect of group×target**				
Study 1	0.16	(1.74, 88.52)	.85	0.02 [0.00,0.06]
Study 2	2.37	(1.36, 461.65)	.11	0.04 [0.00,0.06]
Study 3	0.36	(1, 218)	.55	0.01 [0.00,0.05]

Note. Degrees of freedom for study 1 and 2 are Greenhouse-Geisser corrected.

**Hypothesis 1**: As hypothesized, the ASE was present for both groups, indicated by a significant main effect of target type (see Table 2). Post-hoc t-tests confirmed the expected order of means: angry faces were detected significantly faster than happy faces, *t*(52) = **-**10.04, *p* < .001, *d* = -1.38, 95% CI [-1.76,-0.99], and blue faces, *t*(52) = -5.68, *p* < .001, *d = -*0.78, 95% CI [-1.09,-0.46].

**Fig 2:**
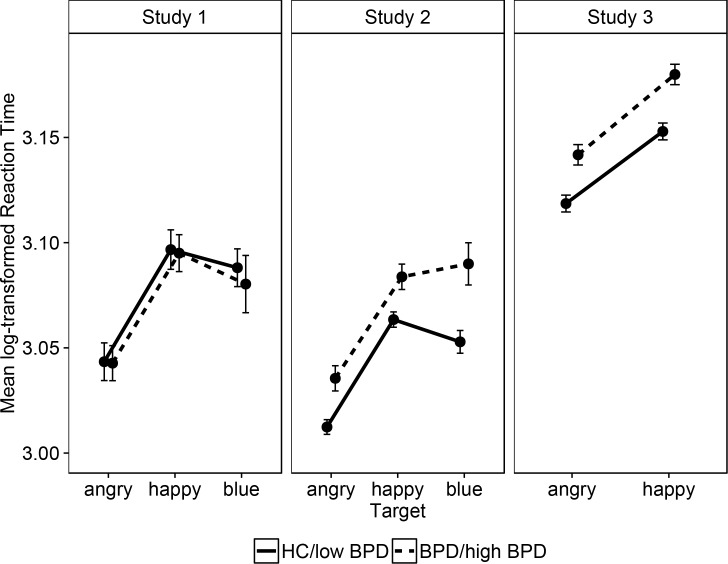
Mean log-transformed reaction times (with 95% CI) for the angry, happy and blue targets conditional on group: Borderline Personality Disorder or high in Borderline features (BPD/ high BPD) vs. healthy controls or low in Borderline features (HC/ low BPD).

**Hypothesis 2:** Contrary to this hypothesis, BPD patients did not show a stronger ASE than HCs, indicated by a non-significant group×target interaction (see Fig 2). Note that all result patterns remained unchanged when repeating analyses (i) without the blue condition, (ii) when dummy-coding for participants’ medication status, (iii) when using non-transformed reaction times, and (iv) when using hitrates as the dependent variable.

## Discussion Study 1

The non-significant group×target interaction points to an equally strong ASE for both BPD patients and HCs. This is in line with a previous study employing the same paradigm but different stimuli [15] and speaks against enhanced emotion detection for angry faces in BPD. However, upon close inspection this finding cannot be taken as conclusive evidence that there is no such effect: With the given sample of n = 53, α = .05 and β = .05, the empirical correlation among repeated measures of r = .80, and a non-sphericity correction of ε = .87, a sensitivity power analysis conducted with g*Power [24] indicated that the interaction effect that can be ruled out within a conventional level of statistical confidence is Cohen’s f = 0.15 (all reported f values in the following analyses refer to the square root of the ratio of effect variance and pooled within groups variances as implemented in g*Power 3.0). Stated simply, this is the smallest effect for which we have sufficient power (1-ß ≥ .95). Consequently, a truly small effect of f = 0.10 or less according to Cohen [25] cannot be excluded with sufficient certainty given the sample size. Thus, we cannot determine whether there really were no differences in emotion detection between the groups, or whether we lacked the sample size to find an existing small effect.

Consequently, it appeared appropriate to conduct a second study with sufficient statistical power to detect even a small effect. To this end, we recruited a large online-sample in which we measured BPD features, a method that has been successfully applied in other studies of BPD [26]. We hypothesized that (1) the general ASE would be replicated and set out to test whether, in this much larger sample, (2) the ASE would be stronger in the group with high BPD scores than in that with low scores.

## Methods Study 2

### Participants

To determine the necessary sample size for ruling out small effects, an a priori power analysis was conducted. To make this replication directly comparable to study 1, we maintained the analytical framework of a repeated measures ANOVA with two groups. The necessary sample size to detect a small effect of f = 0.10, with α = .05 and 1–β = .95 for the interaction term of the repeated measures ANOVA is n = 338. Power analysis was based on the g*Power default value for the correlation among repeated measures of r = .50 and a conservative value of ε = .70. Since we expected drop-out and that some cases would not meet the requirements for adequate data quality, we oversampled by approximately 15%.

Three-hundred-and-ninety-one German speaking participants were recruited via social networks and BPD-related websites such as self-help groups. We ensured that the final dataset contained no repeated participations (identified by an identical IP address, sex, and age) and only retained participants who confirmed to have participated seriously. After applying filters for a minimal trial number of 90% per target type, 342 participants remained in the final dataset. Participants were aged 18 to 60 (M = 25.5, SD = 6.7), 75.7% were female, and 28.9% of participants were recruited from BPD forums or BPD self-help groups.

### Material

BPD features were assessed using the Borderline scale of the Verhaltens-Erlebens-Inventar (VEI) [27], which is the German adaptation of the Personality Assessment Inventory [28]. The scale consists of 24 items measuring the facets identity problems, self-harm, negative relationships, and affective instability, answered on a four point Likert-type scale. The Borderline scale of the VEI has shown a good internal consistency of .84 [27] and validity of the English PAI-BOR has been demonstrated repeatedly [29, 30]. The face-in-the-crowd paradigm was applied in the exact same way as in study 1. Reaction time measurement used client-side JavaScript which provides millisecond accuracy.

### Procedure

While collecting data online, we carefully followed the standards for web-based experiments [31]. At the beginning of the study all participants provided informed written consent after obtaining detailed information about the study procedure. Next, participants completed a personality inventory the results of which are reported elsewhere [32], followed by the VEI. Next, 210 trials of the face-in-the-crowd paradigm were presented; 35 for each target type (angry, happy, blue) and 105 noise trials. Upon completing this task, participants answered several control questions to ensure serious participation.

## Results Study 2

The dataset for Study 2 can be accessed in S2 Dataset. Data were filtered and log-transformed exactly as in Study 1. Next, a group with VEI scores below the clinical cut-off score of 38 [28] and one with participants scoring above were formed. The group below cut-off comprised 203 participants, their VEI scores ranging from 7 to 37, M = 24.3, SD = 7.3, and 139 participants scored above cut-off with scores from 38 to 69, M = 50.1, SD = 8.7. The VEI showed a high internal consistency of α = .92 in this sample.

**Hypothesis 1:** The ASE found in study 1 was replicated across groups. Again, post-hoc t-tests showed angry faces were detected faster than happy faces, t(341) = -22.1, *p* < .001, *d* = -1.20, 95% CI [-1.34,-1.05], and blue faces, t(341) = -9.88, *p* < .001, *d* = -0.53, 95% CI [-0.65,-0.42].

**Hypothesis 2:** As in Study 1, the magnitude of the ASE did not differ between groups (see Table 2; Fig 2). Note that the result patterns remained unchanged when repeating analyses (i) without the blue condition, (ii) using non-transformed reaction times, (iii) using extreme groups of participants with a VEI score ≤ 24 (average rating of maximal 1 on all VEI items) in one group and with a score ≥ 48 in the other (average rating of minimal 2 on all VEI items), (iv) when using hit rates as the dependent variable, (v) in a linear mixed model with the continuous VEI score and target type as predictors.

## Discussion Study 2

As in Study 1, the ASE did not differ between groups. However, for the current sample, G*Power [24] sensitivity analysis for the interaction term indicated that an effect as small as f = 0.07 could be ruled out. The power analysis was based on the observed correlation among repeated measures of r = .74, a non-sphericity correction of ε = .68 and a conventional level of α = .05. Thus, the current sample size implied sufficient power to detect even small effects. Nonetheless, a null-finding such as the present one should be as rigorously subject to replication as a study showing an effect. Thus, to further test the preliminary conclusion that there is no group×target interaction, we decided to replicate our own results in a third study, using another large, web-based sample. Moreover, to ensure that the null finding was not limited to the specific material we used [18], we created a second set of stimulus material with different identities and varying emotional intensities.

## Methods Study 3

### Participants

Analogous to Study 2, we performed an a priori power analysis. The necessary sample size to detect a small effect of *f* = 0.10 with 1–β = .95 and α = .05 was determined as *n =* 198. Assuming a correlation among repeated measures in a similar range as in Study 1 and Study 2 (both r = .75), we adopted the slightly more conservative value of r = .70. We recruited participants in the same fashion as in Study 2 and again oversampled by approximately 15%. Two-hundred-and-twenty-eight participants completed the entire study. All participants provided informed written consent at the beginning of the study and after having obtained detailed information about the study procedure. After applying the previous filter for a minimal trial number, 220 participants remained in the final dataset. Of the remaining participants (aged 18 to 58, *M* = 28.1, *SD* = 8.6), 82.7% were female, and 35.0% were recruited from BPD-related websites.

### Procedure and Material

BPD features were again assessed using the borderline scale of the VEI [27]. The new material for the face-in-the-crowd paradigm was created using Inkscape [23]. Twelve different identities (six female, six male) were constructed in the variants angry, happy and neutral. The intensity of the emotional expression, that is, the degree to which mouths and eyebrows were curved, was varied. To create unsystematic and heterogeneous crowds, neutral faces were randomly selected from the stimulus set per trial and randomly assigned to a position in the 3×3 matrix (see Fig 1 for an exemplary matrix). Each of the 12 angry/happy target faces was presented 3 times, always in a random position in the matrix, within a crowd of randomly selected neutral faces. Hence, participants completed 36 target trials showing angry/happy faces each. The blue baseline stimulus was no longer included, thus participants completed 72 noise trials with neutral matrices. The procedure was identical to that in Study 2.

## Results Study 3

The dataset for Study 3 can be accessed in S3 Dataset.

Data were transformed, filtered and participants were divided into two groups exactly as in Study 2, with 119 participants scoring above and 101 scoring below the clinical cut-off of the VEI. VEI scores ranged from 38 to 68, *M =* 51.5, *SD* = 8.0, in the group above the clinical cut-off and from 5 to 37, *M* = 24.7, *SD* = 7.9, in the group below the cut-off. In this sample, the VEI’s internal consistency was α = .93.

**Hypothesis 1:** The ASE was replicated across groups. Post-hoc t-tests showed that angry stimuli were detected faster than happy ones, *t*(219) = -11.27, *p* < .001, *d* = -0.76, 95% CI [-0.91,-0.60]. As in study 2, the result patterns remained unchanged when repeating analyses (i) using non-transformed reaction times, (ii) using extreme groups of participants with a VEI score ≤ 24 in one group and with a score ≥ 48 in the other, (iii) when using hitrates as the dependent variable, (iv) in a linear mixed model with the continuous VEI score and target type as predictors. **Hypothesis 2:** As previously, there was no significant group×target interaction. G*Power sensitivity analysis for the interaction term indicated that in this sample an effect as small as *f* = 0.07 could be ruled out. Power-analysis based on the empirical correlation among repeated measures of r = .81 and α = .05.

## General Discussion

In a laboratory experiment (Study 1), we tested the hypothesis that BPD patients might differ from HCs in detecting angry faces, indicated by an increased ASE in the face-in-the-crowd paradigm. We did observe a strong ASE, but it did not differ between BPD patients and controls. However, as power analyses revealed, this finding was inconclusive as it may have been the result of the small sample size and thus inherently low statistical power to detect small effects. Hence, we conducted two further studies with large samples and thus sufficient power to rule out even small effects.

For studies 2 and 3, participants were recruited online and BPD features were measured. Participants were divided into two groups based on the clinical cut-off score (one group with, one without clinically significant BPD features). It is important to note that 41% of the sample in study 2 and 54% of the sample in study 3 had clinically relevant BPD features above the clinical cut-off, due to recruitment efforts in the BPD online community. Studies 2 and 3 replicated the pattern observed in study 1: a general ASE was found, but no indication of a group×target type interaction was present, speaking against enhanced anger detection in BPD. Due to the larger sample size, even a small (interaction) effect could be ruled out. Thus, the null findings yield support for the null hypothesis within a conventional level of statistical certainty. Note that study 3 replicated this pattern using heterogeneous stimuli with different identities and varying emotional intensities. The finding is in line with a previous study that employed the same paradigm [15], yet different stimuli, and stands in contrast to the finding by Schulze and colleagues [14].

Regarding limitations of the study, the samples of Studies 2 and 3 must be viewed with some caution. Although measuring BPD features in two large online samples solved the problem of insufficient statistical power, the VEI’s clinical threshold is of course not equivalent to a clinical diagnosis, even though the VEI cut-off is known to correspond very well with diagnoses based on the DSM-IV (82% accordance) [29], (73% accordance) [30]. Also, the influence of third variables such as comorbid diagnoses, substance abuse, medication status etc. could not be controlled for in the web samples.

The results imply that it is necessary to differentiate between emotion detection and emotion categorization in BPD. The first requires the ability to perceive the mere presence of an emotion, whereas emotion categorization requires further cognitive processing. Our experiments repeatedly revealed no difference between patients and controls for the detection of angry faces, which suggests that impairments in emotion processing may only arise later, when faces are evaluated and categorized [6, 7]. A recent study that measured BPD features in a student sample supports the notion of normal emotion detection yet altered (that is, greater) emotion recognition abilities [33].

Our results further have implications for clinical research. It is our view that patient-based, fully-controlled lab-studies and large-scale web-studies could serve as complementary approaches. Indeed, most conclusive evidence will necessarily entail compatible findings from both sources. Given that small-sample, patient-based studies are clearly more common in clinical research, our recommendation is to complement these with large-scale, web-based samples more often and thus test whether effects (or null findings) hold when both the type-I and type-II error are low.

## Supporting Information

S1 Dataset Dataset for study 1. (CSV)

S2 Dataset Dataset for study 2. (CSV)

S3 Dataset Dataset for study 3. (CSV)
